# Impact of treatment time‐related factors on prognoses and radiation proctitis after definitive chemoradiotherapy for cervical cancer

**DOI:** 10.1002/cam4.794

**Published:** 2016-07-15

**Authors:** Eng‐Yen Huang, Hao Lin, Chong‐Jong Wang, Chan‐Chao Chanchien, Yu‐Che Ou

**Affiliations:** ^1^ Department of Radiation OncologyKaohsiung Chang Gung Memorial Hospital, Chang Gung University College of MedicineKaohsiungTaiwan; ^2^ School of Traditional Chinese MedicineChang Gung University College of MedicineKaohsiungTaiwan; ^3^ Department of Obstetrics and GynecologyKaohsiung Chang Gung Memorial Hospital, Chang Gung University College of MedicineKaohsiungTaiwan

**Keywords:** Cervical cancer, concurrent chemoradiotherapy, high‐dose‐rate, intracavitary brachytherapy, overall treatment time, radiation proctitis

## Abstract

This study aimed to investigate the impact of treatment time‐related factors on outcomes and radiation proctitis in patients undergoing concurrent chemoradiotherapy (CCRT) for cervical cancer. From September 2001 to December 2012, 146 patients with stage IIB cervical squamous cell carcinoma treated with CCRT were reviewed from a prospective cohort. Patients who received the same dose (45 Gy) of external beam radiation therapy (EBRT) were included in the analysis (*n* = 125). The same equivalent dose of 2 Gy (EQD2) of high‐dose‐rate intracavitary brachytherapy (HDR‐ICBT) was delivered at either 4 fractions of 6 Gy or 6 fractions of 4.5 Gy. The effects of the overall treatment time (OTT) and interval between EBRT and HDR‐ICBT on the cancer‐specific survival (CSS), local recurrence (LR), and incidence of proctitis were compared. The treatment time‐related factors did not adversely affect the CSS and LR rates. The multivariate analyses did not identify the OTT as an independent factor of CSS (*P *=* *0.839) and LR (*P *=* *0.856). However, OTT ≤56 days (*P *=* *0.026) was identified as the only independent factor of overall proctitis. The 5‐year Grade 2 or greater proctitis rates were 14.9% and 0% (*P *=* *0.001) in patients with the EBRT to ICBT interval ≤5 days and >5 days, respectively. To reduce rectal damage without compromising prognosis, the gap between EBRT and HDR‐ICBT should exceed 5 days in cervical cancer patients undergoing CCRT. Strictly limiting the OTT to 56 days may result in radiation proctitis without improvements in prognosis.

## Introduction

Cancer of the uterine cervix is one of the most common gynecologic cancers worldwide. Radiation therapy (RT) is one of the major treatment modalities for cervical cancer, and it has been associated with an excellent tumor control rate and favorable prognosis. However, adverse effects due to a prolonged overall treatment time (OTT) have been reported in many studies 1, 2, 3, 4, 5, 6, 7, 8, 9. Concurrent chemoradiotherapy (CCRT) via the addition of cisplatin‐based chemotherapy to RT has been demonstrated to provide survival advantages and to increase the biological effective dose of RT. The use of CCRT may avoid the potentially adverse effects of a prolonged OTT in the treatment of cervical cancer. However, a prolonged OTT is associated with pelvic recurrence in patients undergoing CCRT 8, although the opposite has also been recently reported 9. Eifel and Thames 10 reported confounding factors, such as a boost to the tumor/lymph nodes, waiting for the regression of poorly responsive tumors, and additional fractions of intracavitary brachytherapy (ICBT), are associated with a prolonged OTT but were not considered in any other studies. Studying adverse effects of OTT always focuses on decreased local control or survival. However, sporadic reports showed increased late complications in cervical cancer patients with short OTT of radiotherapy alone. This adverse effect of short OTT was not reported for late complications in CCRT patients.

In cervical cancer, studying the effect of OTT is complex because both external beam radiation therapy (EBRT) and ICBT may be involved in this effect 10. Several methods are used to shorten the OTT in cervical cancer: changing the fractionation of radiotherapy, integrating ICBT into the EBRT course, and shortening the gap between EBRT and ICBT if EBRT and ICBT are separated. However, the impacts of OTT shortening on treatment outcome and complications are unclear, especially in patients undergoing CCRT. CCRT increases acute gastrointestinal complications 11, its consequential late effect may play a role in rectal complications 12. In addition, many studies revealed ICBT rectal dose rather than bladder dose correlates corresponding complications. Our original study 13 demonstrated that large fraction size of ICBT increased Grade 2‐4 proctitis but not cystitis. Therefore, we are interested in effect of time‐related factors on rectal complications.

The aim of this study was to investigate the effects of various time‐related factors used to shorten the OTT in patients undergoing CCRT in a subgroup of a previous prospective cohort study with regard to homogenous stage and dose.

## Materials and methods

### Characteristics of patients

We conducted this study based on our previous prospective cohort study 13 to investigate the influence of time‐related radiotherapy factors on the treatment outcomes and complications of patients with cervical cancer. Between August 2001 and September 2012, 146 patients with International Federation of Gynecology and Obstetrics (FIGO) stage IIB squamous cell carcinoma of the cervix were referred to our Department of Radiation Oncology for radiotherapy. Abdominal computed tomography (CT), chest x‐ray, and laboratory studies, including hemoglobin, squamous cell carcinoma antigen (SCC‐Ag), and carcinoembryonic antigen (CEA) were completed before radiotherapy, and patients with CT‐detected para‐aortic lymph node metastases were excluded. The severity of parametrial (PM) involvement was scored based on a previous study 14. In stage IIB disease, this score ranges from 1 to 4.

### Radiation therapy

All patients underwent whole‐pelvic radiotherapy (WPRT) prior to brachytherapy. This radiation was delivered via the four‐field (10‐ or 15‐MV photons) technique. The following standard doses of external beam radiation therapy (EBRT) were administered to the stage IIB patients who underwent CCRT: The WPRT dose was 39.6 Gy/22 fractions, followed by a parametrial boost 5.4 Gy/3 fractions. Therefore, EBRT dose was 45 Gy/25 fractions. Cisplatin‐based chemotherapy was administered to all patients concurrently with radiotherapy. The regimen was either weekly cisplatin or monthly 5‐fluorouracil plus cisplatin. Chemotherapy with weekly cisplatin (40 mg/m^2^) was initiated during the first week of radiotherapy and then was given for six cycles. The monthly regimen was administered as two cycles of 5‐fluorouracil (1000 mg/m^2^/day) on days 2–5 and cisplatin (70 mg/m^2^/day) on day 1 along with RT for five consecutive days at 28‐day intervals.

Patients who completed EBRT underwent high‐dose‐rate intracavitary brachytherapy (HDR‐ICBT) using a remote after‐loading system (microSelectron, Nucletron, the Netherlands) that employed an ^192^Ir source. The gap between EBRT and ICBT was defined as the interval between the last day of EBRT and the first day of ICBT. The details of the HDR‐ICBT procedure have been described elsewhere 13. The typical dose of brachytherapy was 4 fractions of 6 Gy (HDR‐4) or 6 fractions of 4.5 Gy (HDR‐6) at Point A delivered twice per week. These patients received equivalent doses of 2 Gy (EQD2) for the tumor (*α*/*β *= 10) as 70.94 Gy and 71.565 Gy, respectively. The OTT was the sum of EBRT duration, the gap between EBRT and ICBT, and the ICBT duration. The median OTT, EBRT duration, gap between EBRT and ICBT*,* gap between WPRT and ICBT, and ICBT duration were 59, 36, 6, 11, and 16 days, respectively. Therefore, we also studied the effects of EBRT duration (≤35 vs. >35 days), EBRT and ICBT gap interval (≤5 vs. >5 days), WPRT and ICBT gap interval (≤10 vs. >10 days), and ICBT duration (≤15 vs. >15 days) on outcomes. We used a cutoff OTT value of 56 days based on a literature review 4, 8 and the sum of each cutoff value for EBRT duration (35 days), EBRT and ICBT gap interval (5 days), and ICBT duration (15 days).

Because dose and fractionation are closely related to treatment time, the patients receiving a higher EQD2 due to poor tumor response and pelvic lymph node metastasis were excluded in this study but not in the original study 13. Therefore, these 21 patients (14%) belonged to interpatient variation of original study. In total, 125 patients were included in this study. The characteristics of the patients are shown in Table 1. This study was approved by the Institutional Review Board of our Hospital (104‐5422B).

**Table 1:** Characteristics of the patients (*n* = 125)

Parameters	Gap ≤5 days	Gap >5 days	*P* value
Age (years)			0.518
<45	10 (16.4%)	9 (14.1%)	
45–70	44 (72.1%)	51 (79.7%)	
>70	7 (11.5%)	4 (6.3%)	
Pelvic node metastasis		0.325
No	58 (95.1%)	57 (89.1%)	
Yes	3 (4.9%)	7 (10.9%)	
Parametrial score			0.006
1	19 (31.1%)	7 (10.9%)	
2	25 (41.0%)	27 (42.2%)	
3	9 (14.8%)	15 (23.4%)	
4	8 (13.1%)	15 (23.4%)	
SCC‐Ag level (ng/mL)			0.133
<10	44 (72.1%)	38 (59.4%)	
≥10	17 (27.9%)	26 (40.6%)	
Intracavitary brachytherapy		<0.001
6 Gy × 4 (HDR‐4)	42 (68.9%)	19 (29.7%)	
4.5 Gy × 6 (HDR‐6)	19 (31.1%)	45 (70.3%)	
Overall treatment time (days)		<0.001
≤56	43 (70.5%)	11 (17.2%)	
>56	18 (29.5%)	53 (82.8%)	
Cumulative rectal BED (Gy_3_)		0.172
<100	34 (55.7%)	25 (39.1%)	
>100	11 (18.0%)	15 (23.4%)	
unknown	16 (26.2%)	24 (37.5%)	
CCRT courses			0.681
≤3	27 (44.3%)	26 (40.6%)	
>3	34 (55.7%)	38 (59.4%)	

SCC‐Ag, squamous cell carcinoma antigen; BED, biologically effective dose; CCRT, concurrent chemoradiotherapy.

### Follow‐up and statistics

After the completion of radiotherapy, the patients were regularly followed up at the Department of Radiation Oncology or Gynecological Oncology every 2 months in the first year and every 3–4 months thereafter. Local recurrence (LR) or distant metastasis was confirmed by biopsy, physical examination, or imaging studies. The grading of proctitis, enterocolitis, and cystitis was based on RTOG/EORTC toxicity criteria described on a previous cohort study 13. The actuarial rates of cancer‐specific survival (CSS), LR, and complications were estimated with the Kaplan–Meier method, and significant differences between the short and long OTT groups were examined using the log‐rank test. The interval to the last follow‐up was calculated from the last ICBT session. A Cox proportional hazard model with a forward stepwise procedure was used for the multivariate analysis for CSS and LR. The relative risk was represented by the hazard ratio (HR) with a 95% confidence interval (CI). All variables, including the pathology, hemoglobin level, tumor marker level, and OTT, were treated as categorical data. These variables were compared using the chi‐square test or Fisher's exact test. Nonparametric correlations for the EBRT/ICBT duration and EBRT to ICBT interval were calculated using a Spearman correlation. The data were processed and statistically analyzed on a personal computer using SPSS version 17.0 software (SPSS Inc., Chicago, IL) for MS Windows^®^.

## Results

### Association between EBRT to ICBT interval and patient characteristics

An EBRT to ICBT interval >5 days was associated with more advanced PM involvement (*P *=* *0.006). Conversely, the interval was not associated with age, positive pelvic lymph node, CCRT course, SCC‐Ag level, and CCRT course (Table 1). OTT correlated with PM involvement (*P *=* *0.042), EBRT duration (*P *<* *0.001), EBRT to ICBT interval (*P *<* *0.001), and ICBT duration (*P *<* *0.001).

**Figure 1:**
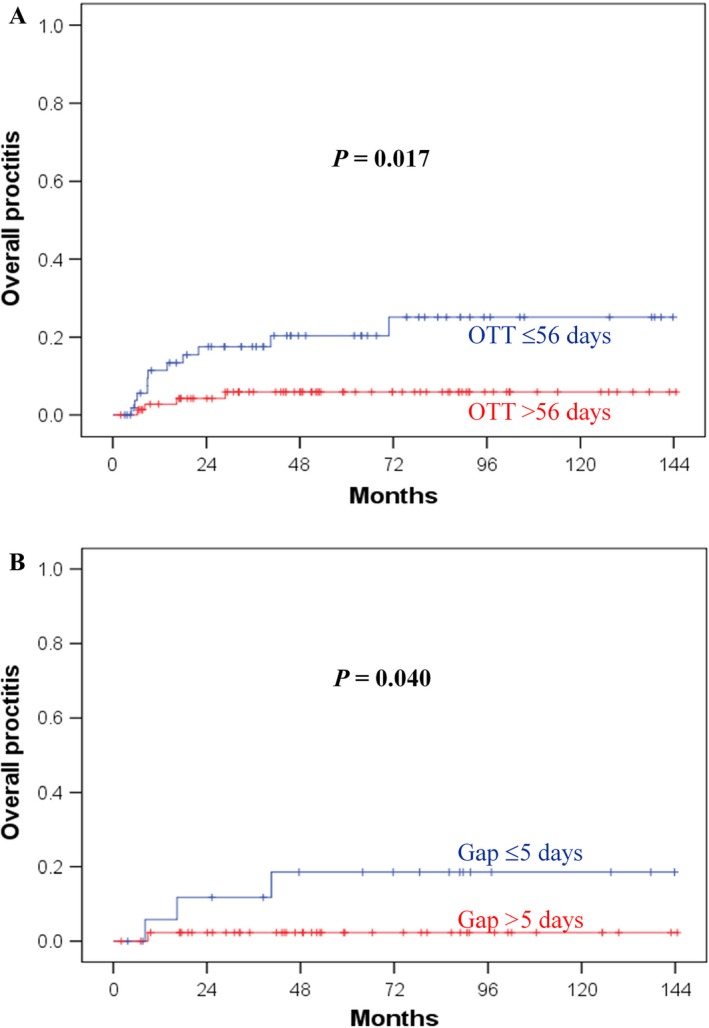
Effect of treatment time on overall proctitis. (A) Overall treatment time (OTT) of 56 days or less increased the risk of proctitis. (B) A gap interval >5 days between EBRT to ICBT could significantly reduce proctitis in patients receiving six fractions of 4.5 Gy ICBT. EBRT, external beam radiation therapy.

**Figure 2:**
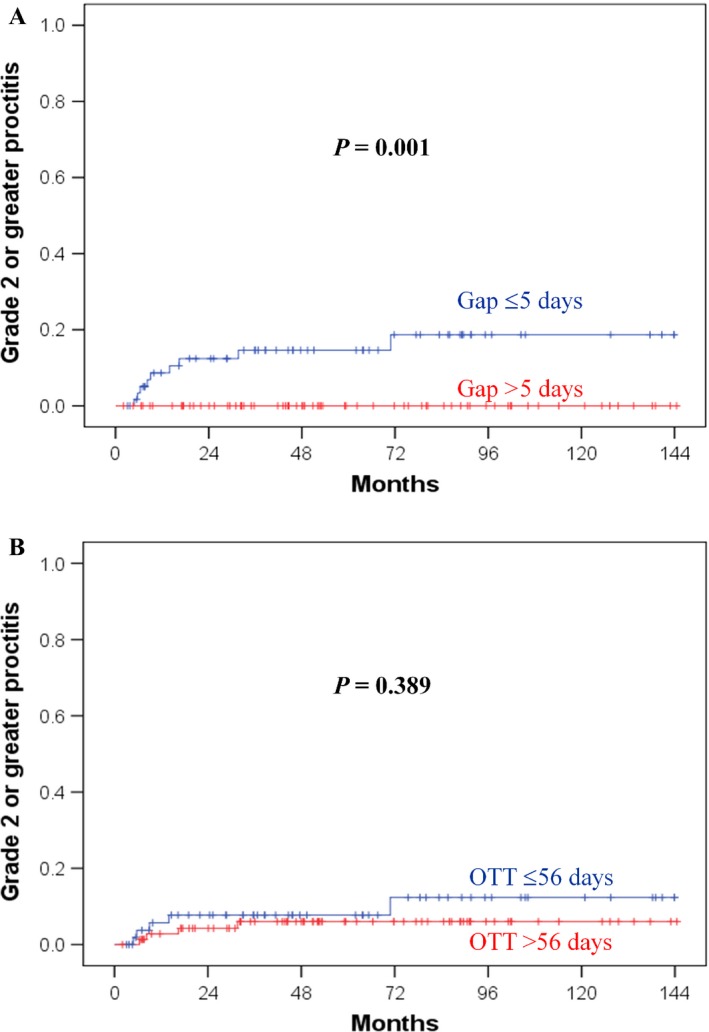
Effect of treatment time on grade 2 or greater proctitis. (A) None of the patients with a gap interval >5 days developed grade 2–4 proctitis. (B) An overall treatment time (OTT) of 56 days or less did not affect Grade 2–4 proctitis.

The median follow‐up time was 83.4 months (range 12–149 months) in the living patients. The 5‐year overall proctitis rates were 19.7% and 7.3% (*P *=* *0.017) (Fig. 1A) in patients with the OTTs ≤56 days and >56 days (Table S1), respectively. The gap interval might correlate with overall proctitis (*P *=* *0.061) (Table S1). In HDR‐6 patients, the 5‐year overall proctitis rates were 18.6% and 2.3% (*P *=* *0.040) (Fig. 1B) for gaps ≤5 days and >5 days, respectively. The multivariate analysis (Table 2) identified an OTT ≤56 days as an independent factor of overall proctitis (*P *=* *0.026). The 5‐year grade 2 or greater proctitis rates were 14.9% and 0% (*P *=* *0.001) in patients with the gap intervals ≤5 days and >5 days (Fig. 2A), respectively. In the HDR‐4 and HDR‐6 groups, the *P* values were 0.048 and 0.023, respectively. An OTT ≤56 days was not a significant factor for grade 2 or greater proctitis (Fig. 2B). The WPRT to ICBT gap >10 days, EBRT duration >35 days, ICBT duration >15 days, and OTT >63 days did not significantly affect radiation proctitis (Table S1). The OTT and gap did not affect enterocolitis and cystitis (Table S2).

**Table 2:** Multivariate analysis of radiation proctitis

Factors	*P* value	HR (95% CI)
Age >63 vs. ≤63 years	0.063	–
HDR‐4 versus HDR‐6	0.417	–
EBRT duration >35 vs. ≤35 days	0.639	–
EBRT to ICBT Gap >5 vs. ≤5 days	0.535	–
WPRT to ICBT WP gap >10 vs. ≤10 days	0.160	–
ICBT duration >15 vs. ≤15 days	0.575	–
OTT >56 vs. ≤56 days	0.026	0.268 (0.084–0.855)
CRBED >100 vs. <100 Gy_3_	0.885	–

HR, hazard ratio; CI, confidence interval; OTT, overall treatment time; ICBT, intracavitary brachytherapy; EBRT, external beam radiation therapy; CRBED, Cumulative rectal biologically effective dose; WPRT, whole‐pelvic radiotherapy.

### Effect of time‐related factors on CSS and LR

The 5‐year CSS and LR rate was 73.3% and 17.1%, respectively. Because a short gap interval contributed to a short OTT and the incidence of radiation proctitis, we investigated the influence of the gap interval and OTT on prognosis. We noted that neither a gap interval >5 days nor an OTT >56 days affected the 5‐year CSS (Fig.S1) and LR rates (Fig.S2). Table S3 shows the results of univariate analysis. Specifically, none of the time‐related factors adversely affected prognosis, including an OTT >56, 63, or 70 days. The multivariate analyses also demonstrated that neither an OTT >56 days nor a gap interval >5 days is an independent factor affecting the CSS and LR rates (Table S4). We show the time‐related factors, prognosis, and grade of proctitis in 11 patients with OTT >10 weeks in Table S5.

## Discussion

The OTT is important in patients undergoing radiotherapy because a prolonged OTT may result in tumor repopulation, as indicated by radiobiological findings 5, 6. Previous studies have examined the role of the OTT in cervical cancer and reported that a prolonged OTT adversely affects the CSS 1 and local control 1, 8. Accelerated radiotherapy regimens using weekly increasing fractions or non‐split courses have demonstrated the effect of the OTT on outcomes in head and neck cancer 15. In cervical cancer, Ohno et al. summarized the results of nonrandomized studies of accelerated hyperfractioned radiotherapy without chemotherapy and noted a grade 3–4 late complication rate of 4.5~37% 16. Although a short OTT may improve outcomes, an increase in the rate complications is a cause for concern when such large fields are irradiated for cervical cancer, especially in patients undergoing CCRT. Our findings support this hypothesis. Because radiotherapy for cervical cancer includes EBRT and ICBT, the time‐related variables that contribute to OTT are complex. Most often, ICBT is started as soon as possible.

Some studies attempting to shorten the OTT also reported increased complication rates 17, 18, 19. Erridge et al. also noted that patients with the shortest OTT had an increased incidence of late morbidity without the involvement of cause‐specific survival 20. The cause may be attributed to ICBT that was performed over the weekend. In this study, the short gap interval may cause radiation proctitis via the superimposed injury of ICBT into the rectum following EBRT. Incomplete repair may be involved in this dose schedule as well as in studies attempting to shorten the OTT. To the best of our knowledge, phase III trials examining the OTT for cervical cancer have not been reported. Therefore, a lack of high‐level evidence likely contributes to OTT effects because OTT is confounded by the tumor response, dose, and stage. The optimal duration of radiation treatment in locally advanced cervical cancer has been derived from retrospective analyses such as the patterns of care studies. Several retrospective studies have reported that the prolongation of the OTT can adversely affect radiation therapy outcomes (Table 3). In patients undergoing RT alone, low‐dose‐rate (LDR)‐ICBT or HDR‐ICBT has been demonstrated to adversely affect OTT prolongation. The optimal cutoff value of OTT varies by study depending on the EBRT dose, dose rate of ICBT, and number of fractions of ICBT. A cutoff of 63 days is consistent with some studies (Table 3), especially in HDR‐ICBT patients. A relatively short cutoff value (42–56 days) has been reported in LDR studies 3, 4, 5, 6, 8. Most patients with positive OTT effects underwent RT alone (Table 3).

**Table 3:** Summary of studies for OTT effects

Author (reference)	No.	CCRT	LDR	Stage stratified	OTT effect (days)	MVA
Fyles 2	830	No	100%	NS	No cutoff	Yes
Petereit 6	209	No	100%	IB + IIA, IIB, III	55	Yes
Lanciano 4	837	No	100%	I, II, III	42, 56, and 70	Yes
Girinsky 3	386	No	100%	NS	52	Yes
Perez 5	1224	No	100%	IB, IIA, IIB	49 and 63	Yes
Chatani 30	216	No	HDR	II, III	42, 49, and 63	Yes
Chen 1	257	No	HDR	IB + IIA	63	Yes
Erridge 20	647	No	100%	NS	No	Yes
Gasinska 7	229	No	100%	NS	60	Yes
GOG120 24	176	Yes	100%	II, III + IV	No	No
GOG165 27	159	Yes	82.3%	II, III + IV	56	No
Song 8	113	Yes	95%	NS	56	Yes
Shaverdian 9	206	No	1.9%	NS	63	Yes
Shaverdian 9	166	Yes	15.1%	NS	No	Yes
Mazeron 29	225	Yes	PDR	NS	55	Yes
Present study	125	Yes	HDR	IIB	No	Yes

NS, Not specified; LDR, low‐dose‐rate; HDR, high‐dose‐rate; PDR, pulsed‐dose rate; MVA, multivariate analysis; OTT, overall treatment time; CCRT, concurrent chemoradiotherapy.

Since 1999, the treatment paradigm for locally advanced cervical cancer has shifted to CCRT 21, 22, 23, 24, 25. An increase in the repopulation of cancer cells has been hypothesized to be due to a prolonged OTT, which reduces local control and decreases treatment efficacy 5, 6. CCRT can improve local tumor control by reducing the accelerated repopulation of tumor cells 26. Only a few studies have discussed OTT in cervical cancer with CCRT (Table 3). Monk et al. reviewed GOG120 24 and GOG165 27 trials and noted inconsistent OTT effects 28. Although more intense parameters of radiotherapy, such as a shorter OTT and higher radiation dose were noted in GOG165 patients, their progression‐free survival was not superior to GOG120 patients. In addition, the rate of acute Grade 3–4 GI toxicities increased in GOG165 patients. Monk et al. suggested that adverse tumor characteristics may frequently confound prolonged RT duration, and OTT may be a proxy variable for other predictors of poor prognosis 28.

Inconsistent OTT effects in cervical cancer patients undergoing CCRT have also been noted in retrospective studies 8, 9, 24, 27, 29. Based on this study, the OTT did affect CCRT patients undergoing HDR‐ICBT. Therefore, concurrent chemotherapy may compensate for the effect of repopulation caused, prolonging the OTT due to radiotherapy. Thus, CCRT may alleviate the adverse effects of OTT prolongation on patients undergoing HDR‐ICBT 1 for the treatment of cervical cancer. However, the OTT effects of LDR‐ICBT patients undergoing CCRT need to be validated in additional studies.

Two critical points should be considered in an OTT analysis. The first point is the stage. In general, a higher EBRT dose is delivered to patients harboring more advanced disease, which consequently prolongs the OTT. Thus, the OTT will be higher in patients with stage IIIB disease than patients with stage IB disease, and this difference has indeed been reported in some studies 1, 4, 5, 6, 9. If a short and long OTT are compared in all patients, without stage stratification, the long OTT group contains more IIIB patients and poor outcomes than the other groups 1, 5, 6, 8. Therefore, patients should be stratified by stage 1, 5, 6 in order to exclude stage as a confounding factor. Without stage stratification, some studies 2, 3, 7, 8, 9 have not been able to clearly define the effect of OTT, despite multivariate analyses. The second point to be considered in an OTT analysis is the radiation dose related to the tumor response. Even for the same stage, the radiation dose may vary based on the tumor response. In general, physicians may prescribe additional EBRT or ICBT doses for poorly responsive tumors 8, thereby also prolonging the OTT. In addition, physicians may wait for further regression, which permits the successful applicator implantation of ICBT. These decisions easily place patients with a potentially poor prognosis in the prolonged OTT group 10.

The causes of treatment prolongation mentioned above are complex 10, 20, 28. In this study, EBRT duration, gap interval, and ICBT duration contributed to the OTT. Complications of chemotherapy may result in interruptions of the radiation schedule, which consequently prolongs the OTT 28. However, the courses of chemotherapy and time‐related duration/interval did not correlate in our study (data not shown). Selecting smaller fractions of ICBT may also prolong the OTT. However, our previous study did not demonstrate an impact of the fraction size on the prognosis 13. This study demonstrated that the gap interval influenced Grade 2‐4 proctitis in both HDR‐4 and HDR‐6 schedules.

The strength of this study is the homogenous dose prescribed to the IIB patients from a previous prospective study 13, which excludes dose as a confounding factor of the OTT. Other differences in patients between original 13 and current study were as follows. First, we excluded non‐SCC patients because these patients had worse prognosis. Second, we excluded dose derivations of protocol such as increase in central dose for poor tumor response of patients who might have worse prognosis. The exclusion rate was 14% for the original protocol which allowed for the interpatient variation. Third, we selected only IIB patients undergoing CCRT. This is the aim of this study. The current report examined the largest sample size (*n* = 125) for IIB patients with CCRT. We did not include IIIB patients because the sample of IIIB patients was too small. Second, we used a multivariate analysis to exclude additional confounding factors. Therefore, this study is the first to investigate the OTT effect for the same stage and dose. Shortening the gap between EBRT and ICBT contributes to radiation‐induced proctitis without improving prognosis. Furthermore, this study excludes patients treated with intensity‐modulated radiotherapy (IMRT) that could be a significant confounding factor in incidence of proctitis.

The limitation of this study is that it was not a randomized trial. Specifically, conducting a prospective study of this hypothesis is difficult, especially when prolonged OTT is an experiment arm, unless ICBT is integrated in the EBRT course. However, this shortened OTT may increase complications, as demonstrated by our results. An OTT ≤56 days (Fig. 1A) and a gap ≤5 days (Fig. 2A) affects the overall and Grade 2‐4 proctitis, respectively. In addition, a gap >5 days (Fig. 1B) reduces overall proctitis in patients with small fraction size of ICBT. To reduce any Grade proctitis, keeping gap >5 days and resulting in OTT within 56–63 days are feasible in HDR‐6 patients. Therefore, we suggest 5 weeks of EBRT, a 1‐week gap, and 3 weeks of a small fraction size of ICBT. Therefore, an OTT of 8–9 weeks may effectively reduce proctitis without adversely affecting prognosis in patients undergoing CCRT. The total EBRT and ICBT doses of this study are lower than those of American Brachytherapy Society (ABS) recommendation. However, Forrest et al. demonstrated 11% of Grade 3–4 complication in HDR‐ICBT patients receiving GOG/ABS recommendation doses and only 80% patients meeting the ABS guidelines for OTT<56 days 31. However, some Japanese groups used lower EQD2/ biologically effective dose (BED) and achieved no inferior outcomes than GOG/ABS recommendation dose 32, 33, 34. The characteristics of our Taiwanese patients may be similar to Japanese rather than US. The 5‐year CSS rate was 73.3% in this study that is compatible with results of clinical trials. In addition, this study did not use modern CT/MR‐guided ICBT to provide accurate dosimetry. Although population of this series is monocentric and very selective, there is a limitation to conduct a multi‐institution study because there were variations of radiation dose and technique within and between institutions. The study is not adequately powered to prove equivalence, which would require a much larger study. Based on our study design (homogeneity of stage, dose, and technique), we encourage another series study of single institution to validate OTT effects.

In conclusion, we suggest a waiting period of more than 5 days before performing HDR‐ICBT in patients undergoing CCRT. Although this strategy prolongs the OTT, it decreases the incidence of grade 2 or greater proctitis without affecting treatment outcomes.

## Conflict of Interest

None of the authors have conflicts of interest to declare related to this study.

## Supporting information


**Figure S1**. No time effects of (A) OTT and (B) gap on cancer‐specific survival rates.
**Figure S2**. No time effects of (A) OTT and (B) gap on local recurrence rates.
**Table S1**. Univariate analysis of radiation proctitis
**Table S2**. Univariate analysis of radiation enterocolitis and cystitis
**Table S3**. Univariate analysis of cancer‐specific survival and local recurrence
**Table S4**. Multivariate analysis of cancer‐specific survival and local recurrence
**Table S5**. Time‐related factors, prognosis, and grade of proctitis in patients with OTT >10 weeks
