# Supplementary material for: Impact of treatment time‐related factors on prognoses and radiation proctitis after definitive chemoradiotherapy for cervical cancer
Source: Cancer Med. 2016 Jul 15;5(9):2205–12. doi: 10.1002/cam4.794 (PMC5055176; doi:10.1002/cam4.794)
Supplement: Supplementary file 1 — Figure S1. No time effects of (A) OTT and (B) gap on cancer‐specific survival rates. Figure S2. No time effects of (A) OTT and (B) gap on local recurrence rates. Table S1. Univariate analysis of radiation proctitis Table S2. Univariate analysis of radiation enterocolitis and cystitis Table S3. Univariate analysis of cancer‐specific survival and local recurrence Table S4. Multivariate analysis of cancer‐specific survival and local recurrence Table S5. Time‐related factors, prognosis, and grade of proctitis in patients with OTT >10 weeks [file CAM4-5-2205-s001.doc]

**Supplement TABLE 1. Univariate analysis of radiation proctitis**

| Time-related factors | Grade 1-4 | P value | Grade 2-4 | P value |  |
| --- | --- | --- | --- | --- | --- |
| EBRT duration >35 vs. ≤ 35 days | 10.9% vs. 13.1% | 0.991 | 9.3% vs. 5.1% | 0.325 |  |
| EBRT to ICBT Gap >5 vs. ≤ 5 days | 7.0% vs. 17.5% | 0.061 | 0% vs. 14.9% | 0.001 |  |
| WPRT to ICBT Gap >10 vs. ≤ 10 days | 11.5% vs. 12.5% | 0.898 | 6.4% vs. 9.9% | 0.698 |  |
| ICBT duration > 15 vs. ≤ 15 days | 8.1% vs. 17.0% | 0.077 | 4.5% vs. 10.6% | 0.124 |  |
| OTT > 56 vs. ≤ 56 days | 7.3% vs. 19.7% | 0.017 | 6.4% vs. 8.1% | 0.389 |  |
| OTT > 63 vs. ≤ 63 days | 11.8% vs. 12.0% | 0.811 | 11.8% vs. 5.6% | 0.514 |  |
| CRBED >100 vs. < 100 Gy_3_ | 16.9% vs. 11.0% | 0.957 | 4.3% vs. 9.2% | 0.367 |  |

*Abbreviations*: OTT= overall treatment time; ICBT= intracavitary brachytherapy; EBRT = external beam radiation therapy; WPRT = whole-pelvic radiotherapy

**Supplement TABLE 2. Univariate analysis of radiation enterocolitis and cystitis**

| Time-related factors | Enterocolitis | P value | Grade 2-4 Cystitis | P value |  |
| --- | --- | --- | --- | --- | --- |
| EBRT to ICBT Gap >5 vs. ≤ 5 days | 13.7% vs. 16.3% | 0.983 | 6.1% vs. 6.9% | 0.928 |  |
| OTT > 56 vs. ≤ 56 days | 14.1% vs. 15.4% | 0.571 | 5.4% vs. 8.2% | 0.346 |  |
| OTT > 63 vs. ≤ 63 days | 13.2% vs. 16.5% | 0.521 | 5.8% vs. 7.4% | 0.674 |  |

*Abbreviations*: OTT= overall treatment time; ICBT= intracavitary brachytherapy; EBRT = external beam radiation therapy

**Supplement TABLE 3. Univariate analysis of cancer-specific survival and local recurrence**

| Parameters | CSS | | |  | LR | | | |  |
| --- | --- | --- | --- | --- | --- | --- | --- | --- | --- |
|  | | *p* value | HR (95% CI) | | |  | *p* value | HR (95% CI) |  |
| PM score (≥3) | | 0.039 | 2.026 (1.023-4.014) | | |  | 0.061 | 2.272 (0.940-5.489) |  |
| SCC ≥ 10 ng/mL | | 0.003 | 3.021 (1.418-6.436) | | |  | 0.078 | 2.490 (0.872-7.109) |  |
| Hb ≥ 10 g/dL | | 0.530 | 1.404 (0.484-4.076) | | |  | 0.743 | 0.806 (0.222-2.930) |  |
| EBRT duration >35 days | | 0.615 | 1.192 (0.601-2.365) | | |  | 0.149 | 1.943 (0.775-4.871) |  |
| EBRT to ICBT Gap >5 days | | 0.980 | 1.009 (0.510-1.997) | | |  | 0.539 | 0.760 (0.315-1.833) |  |
| WPRT to ICBT gap > 10 days | | 0.317 | 1.433 (0.705-2.914) | | |  | 0.981 | 1.011 (0.419-2.440) |  |
| ICBT duration > 15 days | | 0.328 | 0.712 (0.360-1.410) | | |  | 0.511 | 0.746 (0.310-1.792) |  |
| OTT > 56 days | | 0.903 | 1.044 (0.523-2.082) | | |  | 0.856 | 0.922 (0.382-2.224) |  |
| OTT > 63 days | | 0.844 | 0.920 (0.399-2.120) | | |  | 0.828 | 1.119 (0.407-3.080) |  |
| OTT > 70 days | | 0.778 | 1.186 (0.362-3.891) | | |  | 0.575 | 0.563 (0.075-4.205) |  |

*Abbreviations*: SCC-Ag = squamous cell carcinoma antigen; CEA = carcinoembryonic antigen; PM= parametrial; OTT= overall treatment time; ICBT= intracavitary brachytherapy; EBRT = external beam radiation therapy; CRBED = Cumulative rectal biologically effective dose

**Supplement Table 4**

**Multivariate analysis of cancer-specific survival and local recurrence**

| Parameters | CSS | | |  | LR | | | |
| --- | --- | --- | --- | --- | --- | --- | --- | --- |
|  | | *p* value | HR (95% CI) | | |  | *p* value | HR (95% CI) |
| PM score (≥3) | | 0.066 | − | | |  | 0.061 | − |
| SCC ≥ 10 ng/mL | | 0.005 | 2.653 (1.336-5.267) | | |  | 0.204 | − |
| Hb ≥ 10 g/dL | | 0.413 | − | | |  | 0.404 | − |
| Gap > 5 days | | 0.711 | − | | |  | 0.731 | − |
| HDR-6 | | 0.287 | − | | |  | 0.875 | − |
| OTT > 56 days | | 0.839 | − | | |  | 0.856 | − |

*Abbreviations*: SCC-Ag = squamous cell carcinoma antigen; CEA = carcinoembryonic antigen; PM= parametrial; OTT= overall treatment time; ICBT= intracavitary brachytherapy; EBRT = external beam radiation therapy

**Supplement TABLE 5. Time-related factors, prognosis, and grade of proctitis in patients with OTT > 10 weeks**

| Case | EBRT duration | Gap | ICBT duration | OTT | Cancer-related death | LR | Proctitis Grade |  |
| --- | --- | --- | --- | --- | --- | --- | --- | --- |
| 1 | 157 | 4 | 9 | 170 | Yes | Yes | 2 |  |
| 2 | 38 | 21 | 68 | 127 | Yes | No | 0 |  |
| 3 | 83 | 5 | 16 | 104 | No | No | 2 |  |
| 4 | 34 | 13 | 48 | 95 | No | No | 0 |  |
| 5 | 33 | 3 | 56 | 92 | No | No | 0 |  |
| 6 | 63 | 4 | 14 | 81 | No | No | 0 |  |
| 7 | 50 | 8 | 21 | 79 | No | No | 0 |  |
| 8 | 55 | 7 | 14 | 76 | No | No | 0 |  |
| 9 | 36 | 13 | 25 | 74 | No | No | 0 |  |
| 10 | 41 | 10 | 21 | 72 | No | No | 0 |  |
| 11 | 44 | 6 | 21 | 71 | Yes | No | 0 |  |

*Abbreviations*: OTT= overall treatment time; ICBT= intracavitary brachytherapy; EBRT = external beam radiation therapy; LR = local recurrence


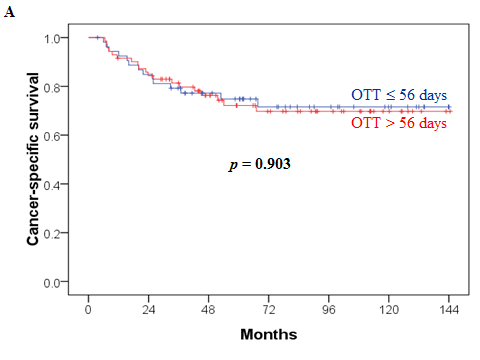

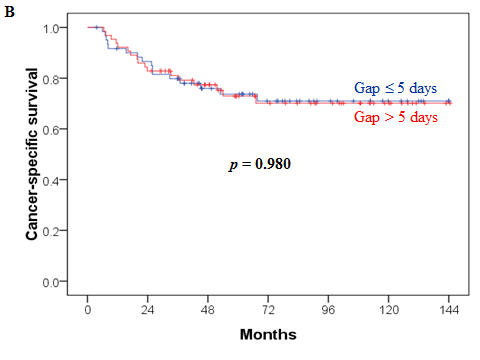


Supplement Fig. 1. No time effects of (A) OTT and (B) gap on cancer-specific survival rates.


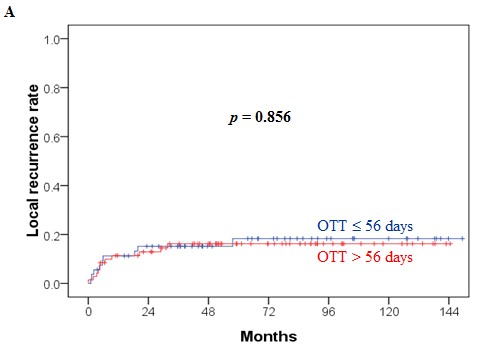

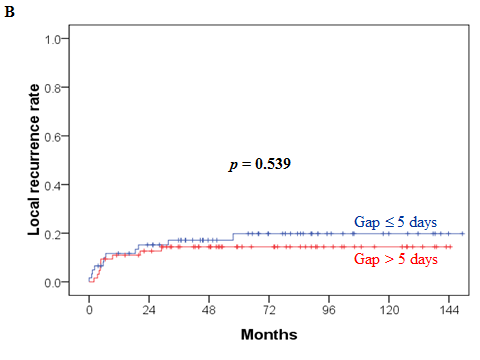


Supplement Fig. 2. No time effects of (A) OTT and (B) gap on local recurrence rates.
